# Independent associations of socioeconomic factors with disability and pain in adults with knee osteoarthritis

**DOI:** 10.1186/1471-2474-14-297

**Published:** 2013-10-17

**Authors:** Rebecca J Cleveland, My-Linh N Luong, Joshua B Knight, Britta Schoster, Jordan B Renner, Joanne M Jordan, Leigh F Callahan

**Affiliations:** 1 Thurston Arthritis Research Center, University of North Carolina at Chapel Hill, Chapel Hill, NC, USA; 2 Department of Health Behavior, Gillings School of Global Public Health, Thurston Arthritis Research Center, University of North Carolina at Chapel Hill, Chapel Hill, NC, USA; 3 University of North Carolina School of Medicine, University of North Carolina at Chapel Hill, Chapel Hill, NC, USA; 4 Thurston Arthritis Research Center and Department of Radiology, University of North Carolina at Chapel Hill, Chapel Hill, NC, USA; 5 Thurston Arthritis Research Center and Departments of Medicine and Orthopedics, University of North Carolina at Chapel Hill, Chapel Hill, NC, USA; 6 Thurston Arthritis Research Center and Departments of Medicine and Social Medicine, University of North Carolina at Chapel Hill, Chapel Hill, NC, USA

**Keywords:** Osteoarthritis, Knee, Pain evaluation, Education, Occupation, Poverty, Social class, Socioeconomic

## Abstract

**Background:**

The purpose of this study is to explore the relationship between function, pain and stiffness outcomes with individual and community socioeconomic status (SES) measures among individuals with radiographic knee osteoarthritis (rOA).

**Methods:**

Cross-sectional data from the Johnston County Osteoarthritis Project were analyzed for adults age 45 and older with knee rOA (n = 782) and a subset with both radiographic and symptomatic knee OA (n = 471). Function, pain and stiffness were measured using the Western Ontario and McMasters Universities Index of Osteoarthritis (WOMAC). Individual SES measures included educational attainment (<12 years, ≥12 years) and occupation type (managerial, non-managerial), while community SES was measured using Census block group poverty rate (<12%, 12-25%, ≥25%). SES measures were individually and simultaneously examined in linear regression models adjusting for age, gender, race, body mass index (BMI), occupational physical activity score (PAS), comorbidity count, and presence of hip symptoms.

**Results:**

In analyses among all individuals with rOA, models which included individual SES measures were observed to show that occupation was significantly associated with WOMAC Function (β =2.91, 95% Confidence Interval (CI) = 0.68-5.14), WOMAC Pain (β =0.93, 95% CI = 0.26-1.59) and WOMAC Total scores (β =4.05, 95% CI = 1.04-7.05), and education was significantly associated with WOMAC Function (β =3.57, 95% CI = 1.25-5.90) and WOMAC Total (β =4.56, 95% CI = 1.41-7.70) scores. In multivariable models including all SES measures simultaneously, most associations were attenuated. However, statistically significant results for education remained between WOMAC Function (β =2.83, 95% CI = 0.38-5.28) and WOMAC Total (β =3.48, 95% CI = 0.18-6.78), as well as for the association between occupation and WOMAC Pain (β =0.78, 95% CI = 0.08-1.48). In rOA subgroup analyses restricted to those with symptoms, we observed a significant increase in WOMAC Pain (β =1.36, 95% CI = 0.07-2.66) among individuals living in a block group with poverty rates greater than 25%, an association that remained when all SES measures were considered simultaneously (β =1.35, 95% CI = 0.06-2.64).

**Conclusions:**

Lower individual and community SES are both associated with worse function and pain among adults with knee rOA.

## Background

Arthritis, and particularly osteoarthritis (OA), is a common chronic disease in the U.S. [1-3] that is a frequent source of chronic pain and a leading cause of disability among older adults [4-6]. The prevalence of OA is expected to increase in the coming decades [7-9], with substantial cost implications for the health care system [10-12]. The knee is the most common joint associated with disability in OA; nearly 600, 000 persons with knee OA opt for total knee replacement every year in the U.S. [13], and the total number of knee replacements is expected to grow to 3.48 million by 2030 [14].

Research has identified individual-level risk factors for OA, such as advancing age and female gender [1]. An individual’s socioeconomic status (SES) may also increase the risk for OA and associated disability. The earliest study exploring the potential role of SES in OA was conducted by Hannan and colleagues [15], which found low educational attainment to be associated with reporting more knee pain and arthritis at any joint. Research has also shown that workplace conditions and occupations requiring strenuous physical movement, such as kneeling or heavy lifting, are associated with increased risk for knee and/or hip OA [16-19]. Johnston County Osteoarthritis (JoCo OA) Project studies have further supported the association of educational attainment and occupation with the prevalence of knee and hip OA in a community sample of Caucasian and African American adults age 45 and beyond [20-22]. Further, individual SES characteristics have also been associated with self-reported pain, physical function and disability among adults with knee and/or hip OA, including educational level [23,24], occupation type [24], employment/retirement status [25], and social class [25].

In addition to individual SES characteristics, community SES factors may also contribute to the risk of developing OA and associated pain and physical function. Research from the JoCo OA Project has shown that both low levels of educational attainment and living in a community with a household poverty rate greater than 25% are independently associated with the risk for radiographic and symptomatic knee OA, even after adjusting for occupation and primary risk factors for knee OA [20]. Similarly, educational attainment and community poverty are associated with the risk for radiographic and symptomatic hip OA [22]. Low education, non-managerial occupation and high poverty rate are associated with less function, and more pain and stiffness in individuals with radiographic and symptomatic hip OA [24].

There have been no known studies to date which have investigated the association between SES factors and functional impairment (or disability), pain and stiffness among those with radiographically defined knee OA. Identification of SES factors that may predict disability among individuals with knee OA could aid clinicians in management of a patient’s OA. The purpose of this investigation is to explore the relationship between physical function, pain and stiffness outcomes with individual SES (education and occupation) and community SES (block group poverty rate) among Caucasian and African American adults with radiographic knee OA (rOA).

### Methods

**Figure 1:**
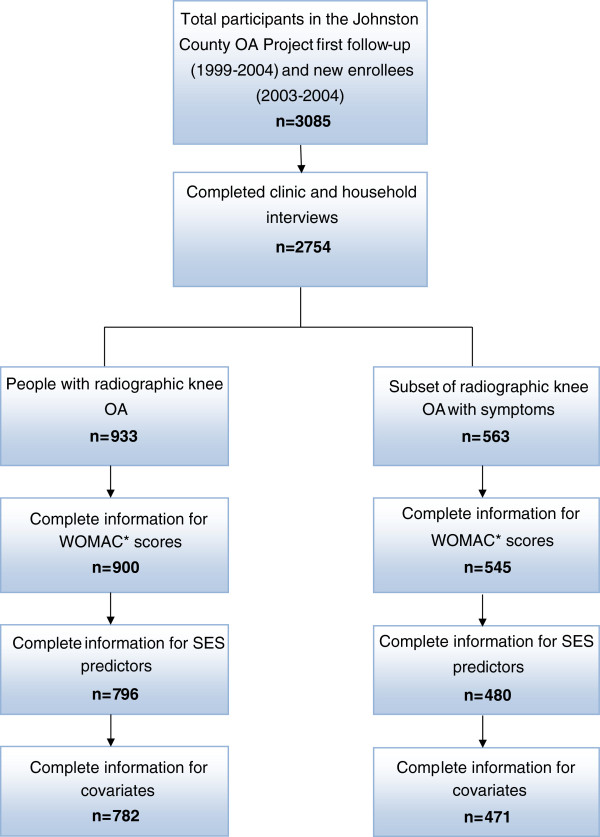
**Participant flow chart.** *WOMAC = Western Ontario and McMasters Universities Index of Osteoarthritis.

The cross-sectional sample investigated in this study was composed of baseline participants in the JoCo OA Project who returned for follow-up in 1999-2004 (n = 1935) or were newly recruited in 2003-2004 (n = 1150). The JoCo OA Project is an ongoing, longitudinal, population-based study of knee and hip OA that includes both rural and urban communities in Johnston County, North Carolina. The design of the JoCo OA Project has been described in detail in a previous publication [26]. A total of 3,085 participants were available for analysis, of which 2,385 had completed all household interviews and clinic visits. Our sample consists of individuals with radiologically confirmed knee OA and a complete WOMAC data (n = 900) (Figure 1). Study parameters were approved by the Institutional Review Boards of both the Centers for Disease Control and Prevention (CDC) and the School of Medicine at the University of North Carolina at Chapel Hill. All participants gave written informed consent at the time of recruitment.

#### Radiographic osteoarthritis assessment

In clinical exams, posterior-anterior radiographs of the knee were obtained and interpreted by a radiologist (JBR) who scored OA on the Kellgren-Lawrence (K-L) scale from 0 to 4 [27]. Presence of rOA was defined as K-L grade at 2 or higher. Both inter-rater reliability and intra-rater reliability were high (weighted inter-rater reliability = 0.86; κappa for intra-rater reliability = 0.89) [28]. A total of 782 participants with complete outcomes, covariates and SES variables had knee rOA (Figure 1).

#### Symptomatic knee osteoarthritis assessment

Individuals with rOA and who had an affirmative response (“Yes”) to the question “On most days, do you have pain, aching or stiffness in your [left/right] knee?” were classified as experiencing symptomatic knee OA. Participants with symptomatic knee OA are a subset of those with rOA (n = 471, Figure 1) who also have pain in the same knee.

### Outcome measures: function, pain and stiffness

The Western Ontario and McMaster Universities Index of Osteoarthritis (WOMAC) questionnaire was used to assess physical functioning, pain and stiffness in hip and knee OA [29,30]. Responses to the WOMAC questionnaire were recorded during the clinical interview. The WOMAC questionnaire includes 24 items grouped into three subscales that assess physical function (17 items), pain (5 items), and stiffness (2 items). Participants are asked to rate the degree of discomfort or difficulty in performing a series of tasks, using a 2-day recall period. Responses are measured on a 5-point Likert-type scale, with higher scores indicating greater pain (0 = none, 1 = mild, 2 = moderate, 3 = severe, 4 = extreme) [29]. In this study, the non-weighted WOMAC Total score (range 0-96) was used along with the Function, Pain and Stiffness subscales.

### Individual SES measures

Educational attainment and occupation were measured to represent individual SES status. Measures of educational attainment have been demonstrated to be a good marker of individual SES among persons with OA [15,31-33]. Participants were asked about their highest school year completed and responses were dichotomized between having less than 12 years of school and having 12 or more years of schooling [referent]. U.S. Census employment classifications were used to categorize participants into one of seven occupational groups based on the job they held for the longest period of time. These groups included: (1) farming, forestry or fishing; (2) management or professional; (3) fabrication or manual labor; (4) precise production, crafting, and repair; (5) service; (6) technology, sales or administration; and (7) military [34]. Job classifications were then dichotomized between non-managerial (groups 1, 3, 4, 5 and 7) and managerial occupations (groups 2 and 6) [referent] according to Census group descriptions. These classifications are used to distinguish occupations that have higher physical demands from occupations that focus on office work, have lower physical demands, and tend to include individuals who have higher SES.

### Community SES measure

We measured community SES based on the community poverty rate, defined as the percent of households that fall below the Census Bureau measure of poverty line [35]. Our measure of community poverty was derived using geocoding technology; the physical address of each study participant at the time of evaluation was linked to U.S. Census-extracted household income data at the block group level and assigned a block group identification code. The census block group averages about 1000 residents and represents the participants’ most immediate community or neighborhood. A participant’s community poverty classification was thereby determined by the block group in which he or she reside, and not by his or her individual circumstances. Participants were categorized based on tertiles of the entire sample resulting in cut-points of 12% and 25%. This community poverty variable was defined as low (less than 12% of population live below the poverty line [referent]), medium (12-25% live below the poverty line), or high (greater than 25% live below the poverty line). Block group poverty rate has been shown to be a good indicator for community-level SES [36,37].

#### Other participant characteristics

Various socio-demographic and clinical characteristics previously associated with knee rOA were collected: age (continuous), Body Mass Index (BMI; weight in kg/height in m^2^) as an obesity indicator (continuous), gender (male [referent] or female), and self-reported race (Caucasian [referent] or African American). An occupational physical activity score (PAS) was calculated based on the participant’s daily report of various occupational activities (standing, walking, squatting and lifting) and the frequency with which he or she performed each activity (0 = never, 1 = seldom,2 = sometimes, 3 = often, and 4 = always), with final scores ranging from 0-16 [21]. Participant hip pain was collected in the same manner as that for knee pain and was assessed with an affirmative response (“Yes”) to the question “On most days, do you have pain, aching or stiffness in your [left/right] hip?” Information was also gathered on the participant’s other non-musculoskeletal health issues to ascertain a total co-morbidity count assessed according to the National Health Interview Survey (NHIS), with some modifications. A comorbidity index of 11 diseases (heart disease, high blood pressure, lung disease, cardiovascular disease, ulcer, liver disease, cancer, anxiety/depression, anemia, diabetes, and kidney disease) was created and defined as the sum of positive responses for individual diseases (range 0-11) [24].

### Statistical analyses

Analyses were carried out based on a complete-case dataset where all subjects had complete information for covariates (Figure 1). Due to a modest amount of missing data for participant occupation (8%) we carried out multiple imputation of missing observations to assess whether the missing data biased our results. We performed multiple imputation of missing occupation using the Markov Chain Monte Carlo method with SAS PROC MI. Results from 10 iterations indicated that there are no meaningful differences in findings from the imputed data compared to a complete-case analysis. Additionally, frequencies of knee disability measures and SES measures were similar for those with complete covariate data and those with at least one missing covariate. Because of these results, and since no individual variables had more than 10% missing values, the results reported are from the complete-case dataset. All statistical analyses were completed in SAS version 9.2 (SAS Institute Inc., Cary, NC).

Within the knee rOA group and the symptomatic knee rOA subgroup, associations between SES variables with each of the WOMAC outcome scores were calculated using ordinary linear regression (OLR). The WOMAC Total distribution was skewed to the right, with relatively high numbers of low values and residuals that did not conform to the assumption of normality. However, tests with proportional odds models, using disability variables assigned to five ordered classes, resulted in findings of significance that are virtually the same as for ordinary regression, as has previously been reported for WOMAC [38]. Therefore, linear regression was retained for ease in interpreting results. Preliminary regressions involved each of the three individual SES variables included in models alone, adjusted for age, gender, race, BMI, hip pain, comorbidity count, and occupational physical activity score. Further multivariable regressions included simultaneous adjustment for all three SES variables and covariates in order to evaluate independent associations between SES variables and WOMAC outcomes.

## Results

### Participants

Characteristics and mean WOMAC scores for individuals with knee rOA and the subgroup with symptomatic knee rOA are presented in Table 1. The sample was largely female (>60%), with a mean age of 67.4 years in the knee rOA group and 66.2 years in the subset with symptomatic rOA. Participants who self-identified as African American comprised 36.6% of the full sample and 40.2% of the symptomatic rOA subgroup. Approximately 36% of the full sample and 37% of the subgroup with symptomatic rOA reported completing less than 12 years of education. The overall sample had a BMI of 32.7, with 58% having a BMI greater than or equal to 30 (a commonly defined marker of obesity) and 65% of the participants in the symptomatic knee rOA subgroup being considered obese. Mean disability scores were generally higher among those within the symptomatic knee rOA subgroup (WOMAC Total = 34.3) as compared to the full knee rOA sample (WOMAC Total = 27.6), indicating worse overall disability.

**Table 1:** **Demographic, SES and WOMAC score characteristics of study participants with rOA and symptomatic**^**§ **^**knee rOA**

**Variable**	**Knee rOA**	**Subset with symptomatic knee rOA**
	**(N = 782)**	**(N = 471)**
Demographic and clinical characteristics		
Age, mean (SD)	67.4 (10.8)	66.2 (11.0)
Female,%	63.6	65.8
African American race,%	36.6	40.2
BMI^‡^, mean (SD)	32.7 (7.58)	33.9 (7.87)
Hip pain,%	42.6	54.6
PAS^¥^ (0-16), mean (SD)	9.40 (3.58)	9.84 (3.54)
Co-morbidity count (0-11), mean (SD)	1.58 (1.38)	1.73 (1.44)
SES characteristics		
Less than 12 yrs education,%	35.5	36.9
Non-managerial occupation,%	39.4	35.9
Poverty, mean (SD)	19.1 (11.5)	19.4 (11.6)
Low (<12%),%	27.0	25.9
Medium (12-25%),%	52.8	52.4
High (>25%),%	20.2	21.7
WOMAC^¶^ Outcomes		
WOMAC^¶^ Function, mean (SD)	19.2 (16.9)	23.6 (16.3)
WOMAC^¶^ Pain, mean (SD)	5.7 (5.1)	7.3 (4.9)
WOMAC^¶^ Stiffness, mean (SD)	2.7 (2.2)	3.4 (2.1)
WOMAC^¶^ Total, mean (SD)	27.6 (23.2)	34.3 (22.1)

^§^Symptomatic knee OA is a subset of those with knee rOA.

^‡^BMI = Body Mass Index.

^¥^PAS = Physical activity score related to occupation.

^¶^WOMAC = Western Ontario and McMaster Universities Index of Osteoarthritis questionnaire.

### Radiographic knee OA analyses

Parameter estimates for covariate adjusted associations of physical function (WOMAC Function), pain (WOMAC Pain), and stiffness (WOMAC Stiffness), and combined physical function, pain and stiffness (WOMAC Total) scores with SES variables (education, occupation type, community poverty) for all participants with knee rOA, are presented in Table 2. Compared with completing 12 or more years of education, completing less than 12 years of education was positively associated with WOMAC Function scores (β = 3.57, 95% CI = 1.25-5.90) and WOMAC Total (β = 4.56, 95% CI = 1.41-7.70) scores. Further, having a non-managerial occupation was observed to have positive associations with three WOMAC scores when compared to having a managerial occupation, including WOMAC Function (β = 2.91, 95% CI = 0.68-5.14), WOMAC Pain (β = 0.93, 95% CI = 0.26-1.59, and WOMAC Total (β = 4.05, 95% CI = 1.04-7.05). Compared with communities with low poverty rates (<12%), living in communities with high poverty rates (>25%) tended to be positively associated with WOMAC Function and Total scores, of borderline statistical significance (both p = 0.05).

**Table 2:** **Associations**^**‡ **^**between SES variables and WOMAC**^**¶ **^**outcomes among participants with knee rOA (n = 782).**

**Effect**	**WOMAC**^¶^**Function β**^**‡ **^**(95% CI)**	**WOMAC**^¶^**Pain β**^**‡ **^**(95% CI)**	**WOMAC**^¶^**Stiffness β**^**‡ **^**(95% CI)**	**WOMAC**^¶^**Total β**^**‡ **^**(95% CI)**
**Individual SES models**				
<12 yrs education^¥^	3.57 (1.25,5.90)**	0.67 (-0.02,1.37)	0.31 (0.00,0.62)	4.56 (1.41,7.70)**
Non-managerial occupation^†^	2.91 (0.68,5.14)*	0.93 (0.26,1.59)**	0.21 (-0.09,0.51)	4.05 (1.04,7.05)**
Poverty rate 12-25%^§^	1.76 (-0.70,4.23)	0.32 (-0.42,1.05)	0.13 (-0.20,0.46)	2.21 (-1.10,5.54)
Poverty rate >25%^§^	3.18 (-0.03,6.39)	0.87 (-0.09,1.83)	0.22 (-0.21,0.65)	4.27 (-0.06,8.60)
**Mutually**^**€ **^**adjusted SES models**				
<12 yrs education^¥^	2.83 (0.38,5.28)*	0.39 (-0.34,1.12)	0.26 (-0.07,0.59)	3.48 (0.18,6.78)*
Non-managerial occupation^†^	1.96 (-0.39,4.30)	0.78 (0.08,1.48)*	0.12 (-0.19,0.44)	2.86 (-0.30,6.02)
Poverty rate 12-25%^§^	1.68 (-0.77,4.13)	0.30 (-0.44,1.03)	0.12 (-0.21,0.45)	2.11 (-1.20,5.42)
Poverty rate >25%^§^	2.81 (-0.38,6.01)	0.77 (-0.19,1.73)	0.19 (-0.24,0.63)	3.78 (-0.54,8.09)

^‡^Adjusted for age, gender, race, body mass index, hip pain, number of comorbidities and occupational and physical activity score (PAS).

^¶^WOMAC = Western Ontario and McMaster Universities Index of Osteoarthritis questionnaire.

^¥^Referent = 12 years or more education.

^†^Referent = managerial occupation.

^§^Referent = poverty rate <12%.

*p < 0.05; **p < 0.01.

^€^All SES measures included in the same model.

Results from multivariable linear regression analyses to assess independent associations with WOMAC outcomes when all three SES variables were included simultaneously are also presented in Table 2. With simultaneous adjustment for all SES measures, the positive associations between low educational attainment and WOMAC Function and WOMAC Total scores remained statistically significant (β = 2.83, 95% CI = 0.38-5.28; β = 3.48, 95% CI = 0.18-6.78, respectively). Further, when compared to having a managerial occupation, having a non-managerial occupation remained significantly associated with higher WOMAC Pain scores (β = 0.78, 95%CI = 0.08-1.48), but was no longer significantly associated with WOMAC Function scores or WOMAC Total scores. The associations with WOMAC scores in individual models for having a non-managerial occupation and a high community poverty rate were further attenuated with the inclusion of all SES measures, so the marginal associations seen were no longer significant.

### Symptomatic knee OA analyses

Statistical analyses identical to those for knee rOA were conducted among the subset of individuals with symptomatic knee rOA (Table 3). When compared to living in a community with <12% poverty, a statistically significant positive finding was observed between participants living in a community with >25% poverty and WOMAC Pain scores (β = 1.36, 95% CI = 0.07-2.65). The associations for WOMAC Function scores and WOMAC Total scores approached statistical significance (p = 0.08; 0.06, respectively). The positive associations of mid-level poverty (12-25%) also approached statistical significance for WOMAC Function scores and WOMAC Total scores (p = 0.10; 0.09, respectively), as did the association between having a non-managerial occupation and WOMAC Pain scores when compared to having a managerial occupation (p = 0.07).

**Table 3:** **Associations**^**‡ **^**between SES variables and WOMAC**^**¶ **^**outcomes among participants with symptomatic knee rOA (n = 471)**

**Effect**	**WOMAC Function β**^**‡ **^**(95% CI)**	**WOMAC Pain β**^**‡ **^**(95% CI)**	**WOMAC Stiffness β**^**‡ **^**(95% CI)**	**WOMAC Total β**^**‡ **^**(95% CI)**
**Individual SES models**				
<12 yrs education^¥^	2.50 (-0.51,5.51)	0.22 (-0.69,1.13)	0.17 (-0.23,0.56)	2.89 (-1.20,6.93)
Non-managerial occupation^†^	2.17 (-0.81,5.15)	0.84 (-0.06,1.74)	0.09 (-0.30,0.49)	3.10 (-0.90,7.11)
Poverty rate 12-25%^§^	2.77 (-0.49,6.04)	0.71 (-0.28,1.69)	0.29 (-0.14,0.71)	3.77 (-0.62,8.15)
Poverty rate >25%^§^	3.80 (-0.47,8.08)	1.36 (0.07,2.65)*	0.31 (-0.25,0.87)	5.47 (-0.28,11.2)
**Mutually**^**€ **^**adjusted SES models**				
<12 yrs education^¥^	1.88 (-1.30,5.03)	−0.08 (-1.00,0.87)	0.14, (-0.28,0.55)	1.94 (-2.30,6.17)
Non-managerial occupation^†^	1.60 (-1.50,4.71)	0.86 (-0.08,1.79)	0.05 (-0.36,0.46)	2.51 (-1.70,6.69)
Poverty rate 12-25%^§^	2.70 (-0.56,5.96)	0.71 (-0.27,1.69)	0.28 (-0.15,0.71)	3.69 (-0.69,8.08)
Poverty rate >25%^§^	3.65 (-0.63,7.92)	1.35 (0.06,2.64)*	0.30 (-0.26,0.86)	5.30 (-0.45,11.1)

^‡^Adjusted for age, gender, race, body mass index, hip pain, number of comorbidities and occupational and physical activity score (PAS).

^¶^WOMAC = Western Ontario and McMaster Universities Index of Osteoarthritis questionnaire.

^¥^Referent = 12 years or more education.

^†^Referent = managerial occupation.

^§^Referent = poverty rate <12%.

*p < 0.05; **p < 0.01.

^€^All SES measures included in the same model.

Results from multivariable linear regressions of function, pain and stiffness simultaneously adjusted for all SES measures are also presented in Table 3. The only statistically significant independent SES predictor of WOMAC scores was seen for community poverty rate. When compared to living in a community with a low poverty rate, living in a community with high poverty rate was positively associated with WOMAC Pain scores (β = 1.35, 95% CI = 0.06-2.64). The elevated associations of high community poverty with WOMAC Function and WOMAC Total remained, although they were no longer statistically significant.

## Discussion

This is the first study to examine community SES and individual SES measures simultaneously as predictors of rOA and corresponding symptoms in a community-based study of African Americans and Caucasians. Our study results indicate that individual SES measures (education and occupation) influence pain and physical function in a cohort of rural, community-dwelling adults with knee rOA. Further, community SES (block group poverty rate) is associated with pain among a subset of individuals with symptomatic knee OA. These data suggest that individuals with knee rOA who are at the highest risk of developing disability and pain are those who have lower SES.

In our analyses, significant associations with pain and physical function remained independently associated with individual and community SES measures for persons with rOA even when the three SES variables were simultaneously introduced as the main explanatory variables in multivariable analyses. Further, our results remained after adjusting for hip pain, which is also a major contributor to disability. In examining the association with educational attainment more specifically, persons with less than 12 years of education were more likely to have worse WOMAC Function and WOMAC Total scores compared to individuals with higher educational attainment, after adjusting for occupation type and poverty. These results are similar to those found in a recent study by Lopez-Olivio et al. which reported that among individuals who underwent knee replacement, those with less than a high school education had worse WOMAC Pain scores and Function scores when compared with those who had at least a high school education [39]. An additional study using data from the National Health and Nutrition Examination Survey I (NHANES I) found that, among individuals self-reporting knee OA, a low educational level was associated with both more severe radiographic findings as well as more pain [40].

We also observed that persons working in non-managerial occupations were more likely to have worse WOMAC Pain scores compared to individuals in managerial positions. Further, persons living in high poverty communities (>25% poverty rate) tended to have worse WOMAC Function and WOMAC Total scores compared to people living in low poverty communities, although not at a level of statistical significance. A recent study reported that adults with chronic knee and/or hip pain who lived in deprived areas were more likely to develop disability [41] while another study reported that patients who lived in areas that were socioeconomically deprived benefitted less from knee replacement surgery than those who did not live in deprived areas [42]. Community poverty may affect those with OA differently, which may lead to greater disability. Communities with high poverty rates often have limited resources including fewer clinics, safe options for public transportation, community centers and safe places to exercise, as well as poorly kept sidewalks and less access to safe streets, all resources that can often contribute to the improvement of function, pain and disability in individuals with OA [43].

Fewer significant associations between SES and pain and disability outcomes were found within the subgroup of individuals with symptomatic knee OA, which may be due to the small subsample size (n = 471). In this subsample, multivariable analyses revealed that living in a high poverty community was significantly associated with WOMAC Pain scores, and associations with WOMAC Function scores and WOMAC Total scores approached statistical significance. We are unsure why we found little association between most SES measures and physical function measures. However, our results for this sub-population are in line with those from another study that failed to find an association for education with disability [44].

Overall these results for the associations of SES on disability among those with knee OA are similar to previous findings between SES and disability among those with OA of the hip [23,24,45-47]. This suggests that a broader rOA relationship may exist between SES measures and physical function, pain and stiffness that is not localized to specific joints, further expanding clinical application of the data. This is especially true for individual SES (education and occupation), but may be equally important for community SES as more sensitive and detailed measures of community SES are tested.

To date, the exact mechanisms of how SES leads to disability remain unclear. While physical features such as deformity and muscle weakness are important aspects, other factors may also have a role in disease progression to disability. A number of cross-sectional studies have demonstrated that psychological [48-50], demographic [51], clinical and biomechanical [52] factors are associated with disability among patients with knee OA. These sociodemographic characteristics may influence health behaviors that lead to OA diagnosis (i.e., seeking medical care) or enhance progression of the disease [53]. Although many of these factors were controlled for in our analyses, it is possible that the associations seen may be due to other circumstances associated with SES, such as lifestyle choices (smoking, diet, obesity, physical activity, etc.), demographic characteristics (age and race), or community and psychological factors (perceived helplessness, social support and perceived discrimination) [54,55]. For instance, obesity and low physical activity levels have both been shown to have both direct and indirect effects on physical function and disability [25,54,56,57]. Further, SES may also have an effect on level of medical care itself. Individuals with lower SES levels often do not have access to affordable medical services and interventions or access to quality healthcare, which are important determinants of health outcomes in OA [58].

There are some limitations to our study that warrant mention. Our study was cross-sectional in design so we can only demonstrate an association and not a causal relationship. Also, our measures of knee disability and functional limitations using the WOMAC instrument, while widely used, are not perfect. However, the WOMAC instrument can detect differences between better vs. worse outcomes when comparing those with mild or moderate disease vs. those with severe knee OA [59]. Additionally, although the measures are still self-reported, WOMAC is designed specifically to measure pain and function of individuals with lower body OA and is among the most sensitive of all instruments used in the assessment of OA of the knee or hip [29] and has been used extensively in observational studies and clinical trials. Further, studies have shown that the WOMAC pain and function subscales exhibit comparable or greater responsiveness to change than corresponding SF-36 subscales [59-61]. Our measure of community SES (block group poverty) is a somewhat crude measure of community influence. However, area-level poverty has been shown to be a good measure for community-level SES and allows for the assessment of the impact of one’s local environment [36,37]. Finally, our outcome measure data were self-reported, which may lead to some overestimation of socioeconomic differences in outcomes, compared with more objective clinical measures of impairment.

Strengths include that we carried out our study in a large community-based cohort of individuals with radiographically-confirmed OA instead of self-reported arthritis, with the inclusion of several SES measures and data for comorbid conditions and hip symptoms. In addition, our study is unique in reporting results among a subset of individuals with symptomatic OA to account for pain. Most studies investigating the effectors of SES on disability have only one measure of SES, usually education. However, here we report results that include three SES measures that we evaluated for independent effects. Importantly, we have included a community-level measure of SES in addition to typical individual-level measures.

## Conclusions

The prevalence of knee OA in the U.S. is very high and is projected to increase due to the growing proportion of older adults in the population, and as a repercussion of the obesity epidemic [9]. With a greater population of individuals suffering from disability as a result of knee OA, it is a matter of public health importance to identify and specifically target features associated with knee disability that can be addressed by clinicians. Although most of the SES measures in our study are not easily modifiable, our results underscore the importance of the clinician’s involvement in the treatment of OA by not only addressing the symptoms, but also by understanding the patient’s context and adapting treatments and interventions based on his or her individual circumstances. Individuals with low levels of education or living in areas with higher poverty levels may need more intensive direction regarding self-management or more help in finding community resources such as weight loss programs that are affordable.

## Abbreviations

BMI: Body mass index; JoCo OA: Johnston County Osteoarthritis; KL: Kellgren-Lawrence; OA: Osteoarthritis; PAS: Physical activity score; rOA: Radiographic osteoarthritis; SES: Socioeconomic status; WOMAC: Western Ontario and McMaster Universities Index of Osteoarthritis.

## Competing interests

None of the authors have financial or personal relationships to disclose. No funds were provided for writing this manuscript.

## Authors’ contributions

RJC participated in analysis and interpretation of data, provided statistical expertise, drafting and critical revision of article. MNL participated in interpretation of data, initial drafting and critical revision of article. JBK participated in drafting and critical revision of article. BS participated in conception and design of study, drafting and critical revision of article, administrative and logistic support. JBR participated in acquisition of data and critical revision of article. JMJ participated in conception and design of study, acquisition of data, interpretation of data and critical revision of article. LFC participated in conception and design of study, analysis and interpretation of data, drafting and critical revision of article, and obtaining of funding. She takes responsibility for the integrity of the work as a whole, from inception to finished article. All authors read and approved the final manuscript.

## Pre-publication history

The pre-publication history for this paper can be accessed here:

http://www.biomedcentral.com/1471-2474/14/297/prepub
